# Diagnostic Approach and Treatment Options for Pediatric Cases of Grisel’s Syndrome Post Otolaryngology Procedure: A Systematic Review

**DOI:** 10.7759/cureus.51739

**Published:** 2024-01-06

**Authors:** Omair H Al-Hussain, Ghadah Al-Hussain

**Affiliations:** 1 Otolaryngology - Head and Neck Surgery, College of Medicine, Imam Mohammad Ibn Saud Islamic University, Riyadh, SAU; 2 Pathology, King Abdulaziz Medical City, Riyadh, SAU

**Keywords:** pediatric, complication, management, diagnosis, grisel syndrome

## Abstract

Grisel's syndrome is an uncommon cervical spine condition marked by non-traumatic rotational subluxation of the atlantoaxial joint. This systematic review aims to collect potential evidence from relevant studies that reported symptoms, diagnostic methods, and management options among pediatric cases of Grisel’s syndrome post otolaryngology procedures, which can aid and guide the diagnosis and management in clinical practice. We conducted both electronic and manual search strategies within the potential databases and included case reports, case series, and articles; however, review papers and correspondence papers were excluded. The post-otolaryngology procedures included adenoidectomy, tonsillectomy, tympanoplasty, cochlear implantation, double opposing Z plasty and pharyngeal flap, and adenotonsillectomy. In this systematic review, we identified and analyzed 20 studies encompassing a total of 24 pediatric patients with Grisel's syndrome following otolaryngology procedures. The patient demographics revealed a fairly even distribution between females (45.83%) and males (50.00%), with ages ranging from 2.5 to 12 years. The most common otolaryngology procedures associated with Grisel's syndrome were adenoidectomy (29.17%) and adenotonsillectomy (33.33%). Clinical symptoms included neck pain (75.00%), torticollis (50.00%), and limited neck mobility (20.83%), while diagnostic confirmation primarily relied on CT scans (50.00%). Treatment strategies varied, with conservative measures being the most frequent choice, followed by surgical interventions in four cases (16.67%). Complications were reported in 20.83% of cases. Due to the rarity of this condition, our findings are limited to case reports only, which may limit the generalizability of results. Grisel syndrome can be effectively managed through conservative treatment, including antibiotics and anti-inflammatory drugs if diagnosed timely. Early diagnosis and prompt management are essential to avoid neurological and fatal complications. This analysis would contribute to improving clinical knowledge and treatment strategies while providing additional insights into this rare condition.

## Introduction and background

Grisel's syndrome is an uncommon cervical spine disorder marked by rotary subluxation of the atlantoaxial joint. It was first reported in the year 1830 by Sir Charles Bell after an atlantoaxial subluxation caused spinal cord compression in a patient with pharyngeal syphilitic ulceration. The disease is named after French physician Pierre Grisel, who reported an additional two cases of inflammation of the nasopharynx that led to the development of this disease in the year 1930 [1,2]. Increased joint ligament laxity, which results in instability, is thought to be the primary cause of this atlantoaxial subluxation. The etiology may involve both inflammations of the soft tissue around the affected area and manipulation of the patient's head and neck during an otolaryngology procedure, which may weaken both the transverse and alar ligaments in patients who already have flexible ligaments [3]. Acute acquired torticollis, atlantoaxial rotatory subluxation, atlantoaxial rotatory fixation, and atlantoaxial rotatory dislocation, among a few others, are terms that have all been used to describe the syndrome. Grisel's syndrome is an atlantoaxial rotatory fixation specifically caused by inflammation or surgery [4].

Grisel's syndrome primarily affects children, with 68% of instances appearing in individuals under the age of 12 years and 90% in patients under the age of 21 years. This disease is significantly associated with head and neck surgical procedures, while upper respiratory infections are considered to be the second most common cause [5]. Following an otolaryngology procedure, Grisel's syndrome is a rare complication that might occur and can lead to severe neurological complications and fatality. A high index of suspicion is essential for an early diagnosis and effective treatment to prevent fatal complications. Any patient who experiences neck pain or torticollis following an otolaryngology surgery should be evaluated for Grisel's syndrome [6]. The diagnostic evaluation is supported by clinical and radiological findings. Patients with painful torticollis, fever, limited range of motion, and pain during reduction attempts should have Grisel's syndrome suspected if there has been no recent trauma, but the patient has a history of upper respiratory infections, head and neck infections, or otolaryngology surgery [7].

The differential diagnoses for Grisel's syndrome that need to be ruled out first include developmental torticollis and traumatic head position. Atlantoaxial rotary subluxation is more common in congenital diseases, including Marfan syndrome and Down syndrome that involve ligamentous laxities. While Kawasaki disease and tuberculosis are two uncommon causes of atlantoaxial rotary subluxation. Case studies in existing literature have associated monopolar suction electrocautery for bleeding control after adenoidectomy with Grisel's disease [8]. Of the 96 cases described by Karkos et al., 48% had an underlying infection [3]. While of the 100 instances reported by Bocciolini et al., 77 were due to infection [9]. Computed tomography (CT) with three-dimensional reconstruction has shown to be a very effective modality for the diagnosis and severity of atlantoaxial rotary subluxation. Although they are rare, neurological complications account for 15% of cases and can be quite serious. These can include quadriplegia, radiculopathy, and mortality from medullary compression-induced respiratory failure. High levels of clinical suspicion should be used to make the diagnosis of atlantoaxial rotary subluxation since treating the condition early on can significantly reduce the risk of serious neurological repercussions [8].

While there have been approximately 90 academic papers published on this subject since 1950, there are currently no definitive guidelines despite the literature reporting several recommendations for diagnosis and treatment. The decision between conservative and surgical treatment for non-traumatic subluxation of the atlantoaxial joint is typically based on the Fielding and Hawkins classification. There have been reports of conservative treatment failure and a high risk of recurrence in cases in the literature when there was a diagnosis delay of more than three weeks following the onset of symptoms. Hence, a timely diagnosis is necessary for a safe and successful course of treatment [10].

Careful clinical assessment and appropriate diagnostic imaging are both necessary for the early diagnosis of atlantoaxial subluxation. The cervical spine's subluxation can be observed on standard X-rays, but the CT scan has a far higher sensitivity for detecting it. Normally, there is a 2-3 mm gap between the dens axis and the posterior portion of the anterior arch of the atlas, but in children, this gap can reach a maximum of 5 mm. A possible atlantoaxial subluxation should be detected if this gap is greater than 5 mm [11]. Early detected Grisel's syndrome is typically treated conservatively, which includes the use of external support or orthoses such as Minerva braces, Philadelphia cervical collars, and sterno-occipitomental immobilizers. For the treatment of primary infection, muscle relaxants, antibiotics, and anti-inflammatory medication are used in conjunction with external assistance. Early diagnosis and treatment are crucial for patients with Grisel's syndrome to have beneficial outcomes. Treatment must begin promptly after the confirmed diagnosis with the aim of reducing and stabilizing the joint. Chronic changes in the transverse and alar ligaments in patients with unreduced atlantoaxial subluxation for longer than three weeks increase the likelihood of recurrence or permanent deformity [12].

In the context of this systematic review, we aim to consolidate existing evidence from relevant studies that detail the symptoms, diagnostic methodologies, and management strategies employed in pediatric cases of Grisel's syndrome following otolaryngology procedures. This analysis seeks to shed further light on this rare condition and contribute to the enhancement of clinical awareness and therapeutic approaches.

## Review

Methods

Definition of Outcomes and Inclusion Criteria

We aimed to investigate diagnostic methods and treatment options among pediatric cases of Grisel’s syndrome post otolaryngology procedures and additionally assess symptoms and complications. Consequently, we included original investigations that recruited pediatric patients who suffered from Grisel’s syndrome post otolaryngology procedures. These post-otolaryngology procedures included adenoidectomy, tonsillectomy, tympanoplasty, cochlear implantation, double opposing Z plasty pharyngeal flap, and adenotonsillectomy. Based on many studies in this field, we have considered pediatric patients between 0 and 15 years old; therefore, all patients above this age were excluded. Moreover, we excluded studies that did not differentiate between pediatric and adult patients in their sample. Review articles and correspondence papers were also excluded from this review. Other exclusion criteria were nonhuman or laboratory studies, nonoriginal investigations or incomplete studies, abstract-only articles, protocols, theses, and articles that were not published in English or with no available English information.

Search Strategy

Based on our determined outcomes, we retrieved the relevant keywords from a brief manual screening within the potentially included studies to design the most suitable search term. We used the following search strategy to search all the relevant databases, including PubMed, Google Scholar, Science Direct, and the Cochrane Library: (Grisel’s syndrome OR Grisel syndrome OR atlantoaxial subluxation) AND (ENT OR otolaryngology OR head & neck OR tonsillectomy OR adenoidectomy OR adenotonsillectomy OR mastoidectomy OR tympanoplasty OR tympanomastoidectomy) AND (children OR pediatric OR Paediatric). Our search strategy was limited to the title and abstract of the search results to utilize all the relevant studies. All of these results were exported to an EndNote library (Clarivate, London, UK) to identify and execute all duplicates between the different searched databases. Furthermore, we manually searched all similar article sections in PubMed and the references of the included studies and relevant studies for possible detection of any missed studies by the main electronic search strategy. All steps of this systematic review were conducted following the Preferred Reporting Items for Systematic Reviews and Meta-Analyses (PRISMA) guidelines.

Screening and Extraction

We performed a double screening strategy; one for screening titles and abstracts and the other for screening full text to maintain high quality in this important process. Two reviewers were involved in this process through a blinded task under the supervision of a senior member, who finished the step by conducting a discussion of the potential differences among the members. After ensuring that all relevant articles were included, an extraction sheet was constructed in an organized way relevant to our aimed outcomes. The sheet consisted of the full title, authors, journal details, study design, abstracts, the decision to include or exclude, and the reason for exclusion.

Quality Assessment

Other information that was extracted from the included studies also included data about the potential risk of bias in these studies. We utilized the Joanna Briggs Institute (JBI) critical appraisal checklist for case reports to assess the quality of all included studies. The checklist consisted of eight questions with the purpose of assessing the methodological quality and reporting of case reports along with the final decision of inclusion of the studies. In applying the JBI checklist, each of the eight domains is evaluated based on specific criteria. "Yes" is assigned when the case report meets the outlined criteria within each domain, demonstrating comprehensive and clear reporting. "No" indicates the absence or inadequacy of essential information or descriptions required by the domain. "Unclear" is designated when the provided information lacks clarity, is ambiguous, incomplete, or does not distinctly fulfill the criteria. This systematic assessment encompasses domains such as patient demographics, presentation of patient history, description of the current clinical condition, detailing of diagnostic tests and results, clear depiction of interventions or treatments, post-intervention clinical condition, identification and description of adverse events, and the provision of takeaway lessons for clinicians.

After systematically assessing the case report across the eight domains outlined in the JBI checklist, the final decision (Yes/No) is reached by considering the cumulative scores. In cases where a "No" or "Unclear" rating was consistently observed across multiple domains, the final decision may lean toward "No" to reflect overall inadequacy in meeting essential reporting standards. Conversely, a consistent pattern of "Yes" ratings across the majority of domains supports a final decision of "Yes," signifying a comprehensive and robust case report that meets the checklist criteria.

Statistical Analysis

Given the anticipated limitations in data homogeneity and the lack of quantitative outcomes across studies, statistical pooling or meta-analysis of results was not performed. Instead, we relied on a descriptive summary of the findings from the individual studies. The results of the analysis of the included studies serve as the primary data source for this systematic review. The synthesis of data from these studies enables us to provide a comprehensive overview of the clinical characteristics, diagnostic methods, and management strategies employed in pediatric cases of Grisel's syndrome following otolaryngology procedures. Categorical data were presented as frequencies and percentages, while continuous data were expressed as means and standard deviations. Fisher's exact tests were employed to assess the significant associations between diagnostic approaches and treatment options for pediatric cases of Grisel's syndrome following otolaryngology procedures.

Results

Search Results

**Figure 1:**
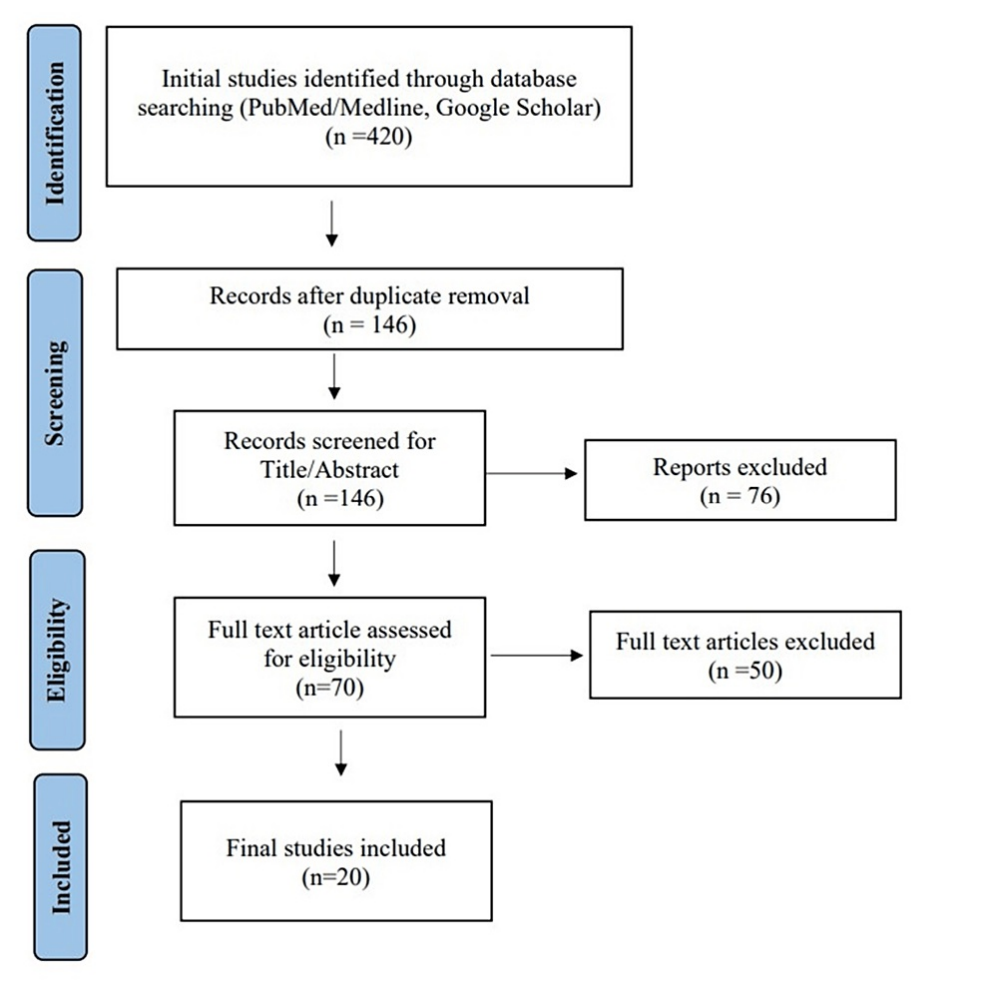
PRISMA flow chart for the selection process to include the relevant studies. PRISMA: Preferred Reporting Items for Systematic Reviews and Meta-Analyses.

By conducting the aforementioned search strategies, we managed to find a total of 420 citations, which were then shortened to 146 after the removal of duplicates. Following title and abstract screening, only 70 citations were eligible for the next steps. Full-text screening showed that only 20 studies matched our inclusion and exclusion criteria. The detailed search strategy and screening are shown in Figure 1.

Results of Quality Assessment

Our assessment of bias for the included studies showed that almost all the studies had good quality and less risk of bias. The included studies showed satisfactory results. The detailed results of the quality assessment according to the JBI critical appraisal tool for case reports are shown in Table 1.

**Table 1:** Quality assessment of the included studies based on the Joanna Briggs Institute (JBI) tool.

Author	Patient’s demographic characteristics clearly described	Patient’s history clearly described and presented as a timeline	Current clinical condition of the patient on presentation clearly described	Diagnostic tests or assessment methods and the results clearly described	Intervention or treatment procedure(s) clearly described	Post-intervention clinical condition clearly described	Adverse events or unanticipated events identified and described	Case report provides takeaway lessons	Inclusion
Pini et al. [7]	Yes	Yes	Yes	Yes	Yes	Yes	No	Yes	Yes
Gross et al. [13]	Yes	Yes	Yes	Yes	Yes	Yes	No	Yes	Yes
Harsh et al. [14]	Yes	Yes	Yes	Yes	Yes	Yes	No	Yes	Yes
Iaccarino et al. [10]	Yes	Yes	Yes	Yes	Yes	Yes	No	Yes	Yes
James et al. [15]	Yes	Yes	Yes	Yes	Yes	Yes	Yes	Yes	Yes
Yi and Chung [16]	Yes	Yes	Yes	Yes	Yes	Yes	No	Yes	Yes
Kim et al. [17]	Yes	Yes	Yes	Yes	Yes	Yes	No	Yes	Yes
Kourelis et al. [18]	Yes	Yes	Yes	Yes	Yes	Yes	No	Yes	Yes
Maglione et al. [19]	Yes	Yes	Yes	Yes	Yes	Yes	No	Yes	Yes
Nakashima et al. [20]	Yes	Yes	Yes	Yes	Yes	Yes	No	Yes	Yes
Ortiz et al. [21]	Yes	Yes	Yes	Yes	Yes	Yes	Yes	Yes	Yes
Serpil et al. [22]	Yes	Yes	Yes	Yes	Yes	Yes	No	Yes	Yes
Park et al. [23]	Yes	Yes	Yes	Yes	Yes	Yes	No	Yes	Yes
Pavlidis et al. [24]	Yes	Yes	Yes	Yes	Yes	Yes	No	Yes	Yes
Reichman et al. [25]	Yes	Yes	Yes	Yes	Yes	Yes	Yes	Yes	Yes
Riney et al. [26]	No	Yes	Yes	Yes	Yes	Yes	No	Yes	Yes
Sakaida et al. [27]	Yes	Yes	Yes	Yes	Yes	Yes	No	Yes	Yes
Sogoba et al. [28]	Yes	Yes	Yes	Yes	Yes	Yes	No	Yes	Yes
Spennato et al. [29]	Yes	Yes	Yes	Yes	Yes	Yes	Yes	Yes	Yes
Miller et al. [30]	Yes	Yes	Yes	Yes	Yes	Yes	No	Yes	Yes

Characteristics of the Included Studies

We included 20 studies that recruited 24 patients and were published between 2011 and 2023 [7,10,13-30]. All the included studies were case reports. Regarding the geographical distribution of the included studies, five studies were of Italian origin, three were of Korean origin, three studies were from the United States, and two studies were from the United Kingdom. India, Mali, Japan, Turkey, Puerto Rico, and Greece were each represented by one study (Table 2).

**Table 2:** Baseline characteristics of the included studies in this review.

Author	Year	Country	Study design	Data collection	Sample size	Gender	Age/mean age	Otolaryngological procedure
Gross et al. [13]	2017	United States	Case report	Prospective	1	Female	4	Adenoidectomy
Harsh et al. [14]	2017	India	Case report	Prospective	1	Female	8	Adenotonsillectomy
Iaccarino et al. [10]	2019	Italy	Case report and literature review	Prospective	2	Male	7 & 9	Adenotonsillectomy & adenoidectomy
James et al. [15]	2017	United Kingdom	Case report	Prospective	2	1 female and 1 male	10 & 14	Mastoidectomy & tympanoplasty
Yi and Chung et al. [16]	2022	Korea	Case report	Prospective	1	Female	8	Tonsillectomy & adenoidectomy
Kim et al. [18]	2011	Korea	Case report	Prospective	1	Male	10	Tympanoplasty & adenotonsillectomy
Kourelis et al. [18]	2015	Greece	Case report	Prospective	1	Female	9	Adenoidectomy
Nakashima et al. [20]	2016	Japan	Case report	Prospective	1	Female	7	Cochlear implantation
Serpil et al. [22]	2020	Turkey	Case report	Prospective	1	Female	6	Double opposing z plasty and pharyngeal flap
Ortiz et al. [21]	2013	Puerto Rico	Case report	Prospective	1	Female	12	Tonsillectomy
Park et al. [23]	2019	Korea	Case report	Prospective	1	Male	6	Adenotonsillectomy
Pavlidis et al. [24]	2015	Italy	Case report	Prospective	1	Male	7	Adenotonsillectomy
Pini et al. [7]	2020	Italy	Case report	Prospective	2	Male	8&9	Adenoidectomy
Reichman et al. [25]	2015	United States	Case report	Prospective	1	Male	4	Adenoidectomy
Riney et al. [26]	2022	United States	Case report	Prospective	1	Not reported	2.5	Tonsillectomy & adenoidectomy
Sakaida et al. [27]	2017	Japan	Case report	Prospective	2	1 male and 1 female	7 & 5	Tympanoplasty & cochlear implantation
Sogoba et al. [28]	2018	Mali	Case report	Prospective	1	Female	5	Tonsillectomy
Spennato et al. [29]	2015	Italy	Case report	Prospective	1	Male	9	Adenoidectomy
Maglione et al. [19]	2021	Italy	Case report	Prospective	1	Female	8.5	Adenotonsillectomy
Miller et al. [30]	2018	United Kingdom	Case report	Prospective	1	Male	11	Adenotonsillectomy

In terms of gender distribution, the cohort comprised 11 female patients (45.83%) and 12 male patients (50.00%). The otolaryngological procedure category illustrated diverse surgical interventions. Notably, seven patients (29.17%) underwent adenoidectomy, while eight patients (33.33%) underwent adenotonsillectomy. Additionally, two patients (8.33%) received cochlear implantation, and one patient (4.17%) each underwent mastoidectomy, double opposing Z plasty and pharyngeal flap, tympanoplasty, and tympanoplasty combined with adenotonsillectomy (Table 3).

**Table 3:** Baseline and clinical characteristics of the patients.

Category	Frequency (%)
Gender	
Female	11 (45.83)
Male	12 (50.00)
Age (mean ± SD)	7.75 ± 2.67
Otolaryngological procedure	
Adenoidectomy	7 (29.17)
Adenotonsillectomy	8 (33.33)
Cochlear implantation	2 (8.33)
Mastoidectomy	1 (4.17)
Tonsillectomy	2 (8.33)
Tympanoplasty	2 (8.33)
Double opposing Z plasty and pharyngeal flap	1 (4.17)
Tympanoplasty & adenotonsillectomy	1 (4.17)

Study Outcome Measures

The primary clinical presentation across the cases was characterized by symptoms such as neck pain, torticollis, and limited neck mobility. Specifically, neck pain or cervical pain was reported by 18 patients (75.00%), while 12 patients (50.00%) presented with torticollis. Other reported symptoms included Cock-Robin position (two patients, 8.33%), fever (four patients, 16.67%), headache (three patients, 12.50%), stiff neck (five patients, 20.83%), cervical pain (four patients, 16.67%), and abnormal head posture (one patient, 4.17%). Diagnostic confirmation of Grisel's syndrome was primarily achieved through CT scans (12 patients, 50.00%), although various diagnostic approaches were utilized, including CT scans combined with MRI (four patients, 16.67%), MRI alone (one patient, 4.17%), X-ray alone (two patients, 8.33%), and combinations of X-ray, CT scan, and MRI (four patients, 16.67%) (Table 4).

**Table 4:** Symptoms, diagnostic approach, and treatment management of Grisel's syndrome.

Symptoms	
Neck pain/cervical pain	18 (75.00)
Cock-Robin position	2 (8.33)
Fever	4 (16.67)
Torticollis	12 (50.00)
Headache	3 (12.50)
Stiff neck	5 (20.83)
Cervical pain	4 (16.67)
Abnormal head posture	1 (4.17)
Diagnostic approach	
CT scan	12 (50.00)
CT scan and MRI	4 (16.67)
MRI	1 (4.17)
X-ray	2 (8.33)
X-ray and CT scan	1 (4.17)
X-ray, CT scan, and MRI	4 (16.67)
Treatment type/management	
Analgesics	1 (4.17)
Antibiotic therapy	2 (8.33)
Antibiotic therapy	1 (4.17)
Antibiotic therapy, cervical orthoses	1 (4.17)
Cervical immobilization, traction	1 (4.17)
Cervical orthoses	3 (12.50)
Cervical orthoses, analgesics	1 (4.17)
Cervical orthoses, anti-inflammatory, antibiotic therapy	1 (4.17)
Manipulation	2 (8.33)
Manual reduction	1 (4.17)
Manual reduction, cervical orthoses	1 (4.17)
Surgical	4 (16.67)
Traction	1 (4.17)
Traction, cervical orthoses	3 (12.50)
Surgical/non-surgical	
Non-surgical	20 (83.33)
Surgical	4 (16.67)
Complications	5 (20.83)

Treatment strategies employed were diverse, with the majority of patients receiving non-surgical interventions (20 patients, 83.33%). Among these, cervical orthoses were commonly used either as a standalone therapy or in conjunction with analgesics or antibiotics. Manual interventions, such as manipulation and manual reduction under anesthesia, were performed in select cases (four patients, 16.67%). Notably, one patient underwent open C1-C3 internal fixation and fusion as a surgical intervention. Complications were reported in five cases (20.83%). A comprehensive overview of study outcome measures is presented in Table 5.

**Table 5:** Summary of the outcomes of the included studies in this review.

Author	Symptoms	Diagnosis	Management	Complications
Gross et al. [13]	Neck pain and left Cock-Robin position of the neck	Type 3 atlantoaxial rotatory subluxation of C1 on C2, with the right C1 lateral mass being more than 5 mm farther forward than the right C2 lateral mass and asymmetric articulation between the C2 lateral masses and the C1 lateral masses were observed on CT scan	Manual reduction of atlantoaxial subluxation under general anesthesia. Full range of motion was regained	None
Harsh et al. [14]	Mild fever associated with neck pain and torticollis toward the right	CT scan of the cervical spine and a confirmatory MRI of the cervical spine revealed lateral displacement of the atlas over the axis with increased distance between the odontoid and the lateral mass on the left side (4.4 mm)	Oral antibiotics (amoxicillin-clavulanic acid), paracetamol, and muscle relaxants along with wearing a cervical collar and bed rest	None
Iaccarino et al. [10]	Painful torticollis and a headache and the second case presented with painful torticollis	Bilateral laterocervical lymphadenopathy with tympanic membrane and external auditory canal hyperemia were revealed on an ENT examination and otoscopy. The cervical X-ray findings were a right lateral flexion of the neck and loss of cervical lordosis. Neck ultrasound scans showed reactive lymph nodes along the jugular chain while a CT scan of the second case disclosed atlantoaxial rotatory subluxation with anterolateral dislocation of C1 and a widened (6.7 mm) distance between the atlas and dens. The atlas was right-rotated by 45° with respect to the axis	Antibiotic therapy along with paracetamol with codeine, prednisone, pridinol mesylate, and chlorhexidine while for the second child, reduction of the rotatory subluxation was accomplished using fluoroscopy under general anesthesia	30° limitation in neck rotation in one case at a 6-month follow-up
James et al. [15]	Painful torticollis and cock robin sign and fixed torticollis in another patient	Dynamic CT scan of one patient demonstrated rotatory deformity of the atlas relative to the axis, which did not change with neck movement while the CT scan of the other patient showed bony cross-fusion between C1 and C2	Examination and manipulation under anesthesia for one child while for other children management options were limited to open reduction and internal fixation, which carried high risk given the child’s co-morbidities. The parents opted to defer surgery in light of the risk. Hence, the neck remained fixed in a rotated position, causing significant restriction	1 patient referred late had a permanent severe positional deformity
Yi and Chung [16]	Neck pain and torticollis	CT and MRI showed type 1 atlantoaxial rotatory subluxation with mild prevertebral soft tissue edema. Asymmetric widening of the gap between the left lateral mass of the atlas and the odontoid process was noted with the narrowing of the gap between the right lateral mass of the atlas and the odontoid process	Cervical halter traction with antibiotics and analgesics	None
Kim et al. [17]	Neck pain and limited range of neck rotation	The cervical radiograph demonstrated an increase in the distance between the anterior surface of the dens and the posterior surface of the C1 tubercle. In the cervical open-mouth view, there was an asymmetry of the odontoid process in relation to the lateral masses of the atlas	Halter traction and Miami brace	None
Kourelis et al. [18]	Painful stiff neck and fever	Neck X-rays displayed only a C2–C3 pseudo-subluxation, with no atlas displacement, suggesting a type I Grisel’s syndrome	A Philadelphia neck brace was placed for 48 h, but as symptoms did not improve, cervical traction was applied next along with ibuprofen and cefuroxime for 7 days	None
Nakashima et al. [20]	Neck pain	A three-dimensional CT scan revealed asymmetry in the atlantoaxial joint and rotation of the atlas with the odontoid process acting as a pivot	Indirect cervical traction and soft collar	None
Serpil et al. [22]	Abnormal head posture, pain, and limitation of neck movements	C1 was rotated 10 degrees left compared with the occiput. There was a 20° rotation between C1 and C2. In the lower cervical vertebrae (C6-C7), this rotational degree was decreasing to 11	A cervical collar (Philadelphia) along with clindamycin	None
Ortiz et al. [21]	Pain over the cervical region and unable to move the neck	Repeated X-rays showed an atlanto-odontoid distance of 6 mm with a diagnosis of C1-C2 subluxation confirmed by CT scan and MRI	Cervical traction followed by fusion and Minerva jacket immobilization	A minor limitation of cervical rotation was noted on the physical examination one year post surgery.
Park et al. [23]	Painful neck movement and head tilted to the right side	CT scan revealed the asymmetry between the dens and the lateral mass of the atlas, and the atlantodental interval was 6.65 mm in the axial image	Cervical immobilization with head halter traction	None
Pavlidis et al. [24]	Cervicalgia, torticollis, and headache	A cervical spine X-ray revealed a right lateral flexion of the neck and loss of cervical lordosis, without bone lesions. Neck ultrasound scans showed reactive lymph nodes along the jugular chain while CT scan and MRI showed atlantoaxial subluxation	Antibiotic therapy with ceftibuten. Paracetamol-codeine, prednisone, pridinol mesylate, and chlorhexidine were added along with the cervical collar	None
Pini et al. [7]	Cervical pain, torticollis, fever, and vomiting, while the second child reported cervical pain and torticollis	MRI showed the presence of C1-C2 rotary subluxation, a fluid collection in the prevertebral space, purulent pharyngeal collection without clear abscess formation, marked edema, and thickening of paravertebral muscles and a CT scan confirmed the atlantoaxial rotary subluxation, with an increase in the atlanto-dens interval of 6 mm, corresponding to a type III Grisel’s syndrome. The CT scan of the second case showed rotatory atlantoaxial subluxation and a hypodense area in the right retropharyngeal tissues	Manual closed reduction followed with Philadelphia brace and antibiotics while the second patient was treated with manual reduction under narcosis followed by Aspen collar	None
Reichman et al. [25]	Fever and worsening torticollis, headache	The diagnosis of Grisel syndrome was considered clinically based on history, physical examination, and radiological evaluations. MRI revealed a small prevertebral abscess anterior to the arch of C1 with surrounding inflammatory changes	Surgery could not be performed long-term antibiotic therapy	Torticollis improved only partially
Riney et al. [26]	Neck pain and stiffness	CT scan showed a head held in axial rotation with soft tissue irregularity in the anterior aspect of the adenoids	Analgesics	None
Sakaida et al. [27]	Painful torticollis and neck fixed to one position while the second case reported neck pain and diminished range of motion	CT scan of the first patient showed that the C1-C2 joint was locked, with C1 rotated toward the left and tilted to the right while the diagnosis for the second patient was also confirmed through CT scan findings	Traction and soft cervical collar while the second case was only treated with the soft cervical collar	None
Sogoba et al. [28]	Swelling and pain in the neck with deviation to one side and restricted range of motion	CT scan showed rotary subluxation at C1/C2 type I according to the Fielding and Hawkins atlantoaxial rotary subluxation classification, with a lateral shift of the dens toward the right	Analgesic and soft cervical collar	None
Spennato et al. [29]	Painful torticollis occurred with mild rotation of the head to the right	CT scan confirmed the atlanto-axial rotatory subluxation with anterolateral dislocation of C1 and showed a clearly widened distance between the atlas and dens, measuring 6.7 mm. The atlas was right-rotated 45 degrees with respect to the axis	Open C1-C3 internal fixation and fusion under general anesthesia	30 degrees limitation of the rotation of the head
Maglione et al. [19]	Neck pain and stiffness along with limited flexo-extensory and rotatory movements	CT scan showed atlantoaxial rotatory subluxation	Cervical collar, anti-inflammatory, and antibiotic treatments	None
Miller et al. [30]	Neck stiffness and abnormal head posture	CT scan revealed unilateral rotatory atlantoaxial subluxation, with more than 75% of the right lateral mass of C1 displaced anteriorly and likely causing damage to the anterior spinal ligament	Manipulation under anesthesia	None

Table 6 provides an in-depth comparison of otolaryngology procedures in relation to diagnostic approaches and symptom presentations of Grisel's syndrome. It elucidates the distribution of procedures across different diagnostic approaches and symptoms, accompanied by frequencies and percentages. Notable findings include the prevalence of specific procedures within particular symptom categories, such as adenoid and tonsil surgery being more frequent in cases involving neck pain or cervical pain and Cock-Robin position. Middle ear procedures, including tympanoplasty, are predominant among cases associated with abnormal head posture and complications. Cervical orthoses emerge as a common treatment modality, either as a standalone approach or in combination with other therapies like analgesics or anti-inflammatory and antibiotic regimens. Manual interventions, such as manipulation and manual reduction, are observed in select cases, often in conjunction with cervical orthoses. Interestingly, cases involving middle ear procedures show a higher propensity for the use of "Cervical Orthoses" and "Traction, Cervical Orthoses" as part of the management strategy.

**Table 6:** Diagnostic approaches and treatment options for pediatric cases of Grisel's syndrome following otolaryngology procedures.

Variable names	Categories	Adenoid and/or tonsil surgery (n, %)	Middle ear procedures (n, %)	Other combined procedures (n, %)	Fisher exact p-value
Diagnostic approach	CT scan	7 (41.18)	4 (80.00)	1 (50.00)	0.610
	CT scan and MRI	3 (17.65)	1 (20.00)	0 (0.00)	
	MRI	1 (5.88)	0 (0.00)	0 (0.00)	
	X-ray	1 (5.88)	0 (0.00)	1 (50.00)	
	X-ray and CT scan	1 (5.88)	0 (0.00)	0 (0.00)	
	X-ray, CT scan, and MRI	4 (23.53)	0 (0.00)	0 (0.00)	
Neck pain/cervical pain	No	4 (23.53)	2 (40.00)	0 (0.00)	0.593
	Yes	13 (76.47)	3 (60.00)	2 (100.00)	
Cock-Robin position	No	16 (94.12)	4 (80.00)	2 (100.00)	0.507
	Yes	1 (5.88)	1 (20.00)	0 (0.00)	
Fever	No	13 (76.47)	5 (100.00)	2 (100.00)	0.680
	Yes	4 (23.53)	0 (0.00)	0 (0.00)	
Torticollis	No	8 (47.06)	2 (40.00)	2 (100.00)	0.497
	Yes	9 (52.94)	3 (60.00)	0 (0.00)	
Headache	No	14 (82.35)	5 (100.00)	2 (100.00)	0.664
	Yes	3 (17.65)	0 (0.00)	0 (0.00)	
Stiff neck	No	12 (70.59)	5 (100.00)	2 (100.00)	0.550
	Yes	5 (29.41)	0 (0.00)	0 (0.00)	
Cervical pain	No	13 (76.47)	5 (100.00)	2 (100.00)	0.680
	Yes	4 (23.53)	0 (0.00)	0 (0.00)	
Abnormal head posture	No	16 (94.12)	2 (0.0%)	2 (100.00)	1.000
	Yes	1 (5.88)	0 (0.00)	0 (0.00)	
Complications	No	13 (76.47)	4 (80.00)	2 (100.00)	1.000
	Yes	4 (23.53)	1 (20.00)	0 (0.00)	
Treatment categorization	Only analgesics	1 (5.88)	0 (0.00)	0 (0.00)	0.953
	Only antibiotic therapy	3 (17.65)	0 (0.00)	0 (0.00)	
	Antibiotic therapy, cervical orthoses	1 (5.88)	0 (0.00)	0 (0.00)	
	Cervical immobilization, traction	1 (5.88)	0 (0.00)	0 (0.00)	
	Cervical orthoses	1 (5.88)	1 (50.00)	1 (20.00)	
	Cervical orthoses, analgesics	1 (5.88)	0	0	
	Cervical orthoses, anti-inflammatory and antibiotic	1 (5.88)	0 (0.00)	0 (0.00)	
	Manipulation	1 (5.88)	0 (0.00)	1 (20.00)	
	Manual reduction	1 (5.88)	0 (0.00)	0 (0.00)	
	Manual reduction, cervical orthoses	2 (11.76)	0 (0.00)	0 (0.00)	
	Manual reduction, cervical orthoses	1 (5.88)	0 (0.00)	0 (0.00)	
	Surgical	3 (17.65)	0 (0.00)	1 (20.00)	
	Traction	1 (5.88)	0 (0.00)	0 (0.00)	
	Traction, cervical orthoses	0 (0.00)	1 (50.00)	2 (40.00)	

These comprehensive findings shed light on the nuanced clinical characteristics, diagnostic methods, and treatment modalities employed in pediatric cases of Grisel's syndrome following otolaryngology procedures.

Discussion

Grisel's syndrome, characterized by atlantoaxial rotatory subluxation, is a condition that predominantly affects children but can also manifest in adults. Our systematic review aimed to provide insights into this rare yet clinically significant condition, particularly in the context of otolaryngological procedures among the pediatric population.

The findings of our review demonstrated that neck pain, torticollis, and limited neck mobility were the primary clinical manifestations reported, followed by Cock-Robin position, fever, stiff neck, and abnormal head posture. Diagnostic confirmation of Grisel's syndrome was primarily achieved through CT scans; however, diverse diagnostic modalities were used, including a combination of CT scan and MRI, MRI alone, and combinations of X-ray, CT scan, and MRI. Barcelos et al. stated that the three-dimensional CT of the craniocervical transition is the gold standard diagnostic examination for atlantoaxial rotatory subluxation since the head's rotational position makes it impossible to take a high-quality radiograph. Authors further described that asymmetry and deletion of the C1-C2 articular surfaces, an increase in the lateral C1 mass anteriorly displaced, and a decrease in the contralateral C1 mass are among the radiographic findings in transoral occurrence [31].

Similarly, Das et al. described that an increased atlanto-odontoid gap may be seen on a lateral plain radiograph of the neck. In children, an atlanto-odontoid gap over 5 mm is noteworthy. A cervical CT scan is sensitive to early subluxation and provides a detailed picture of the joints. The abnormality can be seen in a three-dimensional reconstruction of the images, which aids in planning the best management strategy. When surgical intervention is intended to rule out neurological involvement, MRI is recommended [32]. The distance ranged from 4 mm to 6.7 mm in this review.

Symptoms including torticollis, neck pain, and head tilt are usually unspecific, and a diagnosis is suggested by the clinical context, presented after a few days or weeks of otolaryngology procedure or infection. A combination of clinical examination and proper radiographic evaluation is necessary for the diagnosis of Grisel's syndrome. Radiological findings eliminate potential diagnoses such as para pharyngeal abscess, still-active ear, nose throat infection, and neurological and traumatic causes. Neurologic complications are more likely to occur with a delayed diagnosis [33-35]. While Beyazal et al. reported that the patient's medical history and clinical symptoms are crucial indicators of the diagnosis, neck pain is the commonly reported symptom that worsens with attempted motion. The Cock-Robin posture, in which the head is held stationary with the chin moved away from the affected side and the head inclined, is a pathological position where the nuchal pain extends to the head or ears. There have also been reports of trismus, diminished hearing, and facial asymmetry with restricted cervical motion, particularly in untreated cases. Usually, there is a history of an upper respiratory illness or an otolaryngological procedure that led to the condition. The diagnosis of an atlantoaxial rotary subluxation is based on three clinical symptoms. The first is a palpable deviation of the axis' spinous process in the same rotational direction as the head. C2 deviates to the opposite side during a typical head rotation, ipsilateral sternocleidomastoid muscle spasm, and the third indication is the inability to turn the head in the opposite direction of the injury beyond the midline [36]. These findings are in accordance with our results, as our review results demonstrate that almost all of the patients reported neck stiffness and pain along with the Cock-Robin sign, while only three cases reported fever additionally.

During the onset of pharyngitis, torticollis may develop spontaneously, or it may follow minor neck trauma. The syndrome has been linked to rheumatic diseases, cervical osteomyelitis, tonsillectomy, adenoidectomy, choanal atresia repair, and mastoidectomy, as well as other rheumatic and surgical conditions. Patients affected are often between the ages of five and 12 years old, and there is no predominant sex. Yet, the spectrum of the disease is documented from infancy to the seventh decade of life. The severity of the atlantoaxial joint subluxation leads to these varied complications [9]. Our results exhibit that the otolaryngology procedures performed among our cases were primarily adenoidectomy tonsillectomy, mastoidectomy, tympanoplasty, and adenotonsillectomy, while cochlear implantation and Z-plasty were performed in only one case each. The age of our study cases varied from 2.5 to 12 years of age.

Yu et al. described that tonsillectomy and adenoidectomy are quite prevalent surgical procedures performed. These operations seldom result in complications, which carry about the same risk as those associated with general anesthesia alone. Due to the nature of the surgical operation, postoperative complaints from patients, including neck pain and stiffness, sore throat, dysphagia, and otalgia, are not uncommon. These nonspecific symptoms and indications, though, are not always normal findings and may result in more severe diseases such as osteomyelitis, abscess, cervical subluxation, and bacterial meningitis. If not detected in time, uncommon and rare complications from these treatments can have detrimental consequences. Unfortunately, after head and neck surgeries, the majority of signs and symptoms are general and frequent, delaying diagnosis. The length of time between diagnosis and complications is directly correlated. Moreover, there may be a varying amount of time between the triggering incident and the beginning of symptoms, making the diagnosis more challenging. A strong index of suspicion is needed to identify Grisel’s syndrome in addition to other uncommon complications following adenotonsillectomy [37]. Similarly, Kraft and Tschopp further confirmed that after an adenotonsillectomy, postoperative neck issues are a frequent observation. However, in cases of prolonged neck pain and stiffness, non-traumatic subluxation of the atlantoaxial joint should be taken into account [38]. In our study, we observed that among patients presenting with neck pain or cervical pain and Cock-Robin position, adenoid and tonsil surgery were the most common otolaryngological procedures performed while abnormal head postures and associated complications were more predominantly reported with middle ear procedures, including tympanoplasty.

Historically, various theories have been proposed to explain the etiology of Grisel's syndrome. One hypothesis suggests that muscle spasms may lead to subluxation, while the more recent two-hit hypothesis posits that hyperemia following infection or trauma can decalcify the anterior arch of C1 and render the transverse ligament flexible [8]. Rami et al. further explained in their study that the vascular plexus that provides drainage for the posterosuperior pharyngeal region is the culprit. The pharyngovertebral vein connects the periodontoid plexus to the posterior nasopharyngeal veins. This plexus, which lacks a lymph node, allows any infectious embolism to move from the superior pharyngeal area to the upper cervical joints, offering an anatomical explanation for the atlantoaxial hyperemia observed in Grisel's syndrome [39].

**Figure 2:**
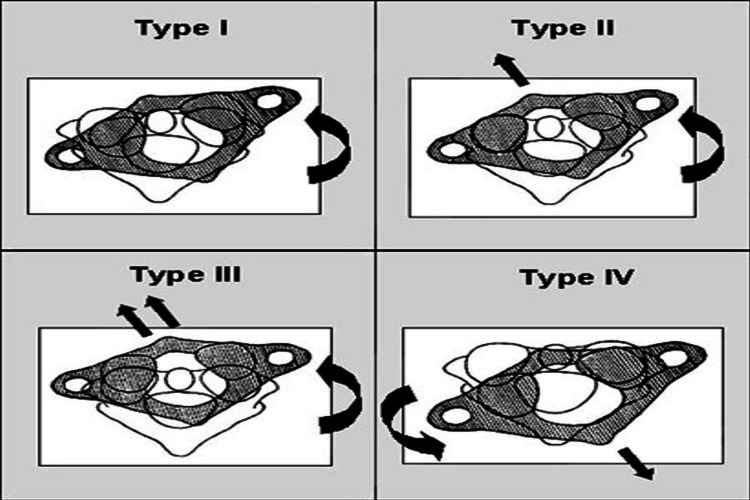
Classification of Grisel's syndrome by Fielding and Hawkins. Type I: rotatory fixation without anterior displacement of the atlas (≤3 mm). Type II: rotatory fixation with anterior displacement of the atlas of 3-5 mm. Type III: rotatory fixation with anterior displacement of the atlas of 5 mm. Type IV: rotatory fixation with posterior displacement of the atlas. Adapted from: Bocciolini C, Dall'Olio D, Cunsolo E, Cavazzuti PP, Laudadio P: Grisel's syndrome: a rare complication following adenoidectomy. Acta Otorhinolaryngol Ital. 2005, 25:245-9.

Atlantoaxial joint rotatory subluxations were categorized by Fielding and Hawkins into four different categories. The atlas's anterior displacement on its axis at the sagittal plane and the rotational center serves as the basis for this classification. Delay in diagnosis is attributed to types I and II. In type I lesions, the transverse ligament, which serves as the atlantoaxial joint's primary stabilizer, is intact. According to the Fielding and Hawkins categorization, type II, III, and IV lesions are thought to involve the transverse ligament. Type I is rotatory fixation in which the atlas is not anteriorly displaced; type II is rotatory fixation in which the atlas is anteriorly displaced by 3-5 mm; in type III rotatory fixation, anterior displacement is greater than 5 mm; and in type IV, rotatory fixation is posteriorly displaced [40,41]. A pictorial representation of the classification of Grisel’s syndrome is illustrated in Figure 2. Most of the cases in our review were of type I, while a few were of types II and III.

For the management of Grisel's syndrome, there is currently no proven gold standard. In the literature, the Fielding-Hawkins categorization is frequently used to outline treatment plans for Grisel’s syndrome. Regular clinical follow-up is necessary for each type of Grisel’s syndrome to detect any potential symptom development timely. It is advised to have a neurological evaluation every two weeks to ensure early identification of any clinical developments. When there are no neurological abnormalities, conservative care is the first line of treatment. For type 1, treatment options include anti-inflammatory drugs, muscle relaxants, and immobilization with a soft collar. For type 2, reduction and immobilization with a rigid cervical collar are recommended. Early, non-aggressive physiotherapy administered below the pain threshold is recommended to treat ankylosis. Type 3 necessitates cervical traction, bed rest, and a period of first-line cervical immobilization. The degree of the beneficial effect of reduction followed by traction is dependent upon the duration of symptoms prior to treatment. Surgery with an arthrodesis is indicated only in the case of failure of conservative treatment or as a first-line emergency in the case of neurological complications [35]. Spennato et al. suggested that in the case of surgery, rigid C1-C2 or C1-C2-C3 fixation is a simple and effective surgical technique, even in children, in cases of failure of conservative treatment, or cases of delayed diagnosis because it immediately provides spinal stability in all planes at the atlantoaxial complex, avoiding the need for protracted rigid external bracing [29]. Our results reveal that nine patients were treated with conservative treatment; two patients among these also underwent cervical halter traction additionally, and cervical traction was performed independently in five patients. Manipulation and manual reduction under anesthesia were performed in four patients. While surgery involving open C1-C3 internal fixation and fusion was necessitated in one patient only.

There are variations in the management strategies for this syndrome. For types I and II, several authors advocate a conservative approach, while types III and IV demand a more invasive strategy. Another treatment method, type I soft collar, type II hard collar, type III close fixation with halo, and type IV open fixation with halo, was also proposed. However, these algorithms may be modified in each situation, and patients should be managed and regulated on an individual basis. Appropriate treatment of the infectious condition, repair of bone deformities, and prevention of complications are the three pillars of the effective management of Grisel’s syndrome [42]. Bocciolini et al. recommended two measures to minimize the occurrence of Grisel’s syndrome post otolaryngology procedure: children under general anesthesia should not have their heads rotated or extended excessively, and following surgery, a rollboard should be used to transport the patient from the operating table to the hospital bed to reduce cervical trauma. The inefficient management of Grisel’s syndrome may lead to permanent neck deformity and even necessitate surgical fusion. According to reports in the literature, in almost 15% of the patients, neurological issues may result in conditions ranging from mild paresthesia and clonus to quadriplegia, acute respiratory failure, and even death [9]. Three cases in our included studies reported complications of the limited range of motion; torticollis could be partially improved in one case, and one case had permanent neck deformity due to delayed treatment due to late diagnosis and referral. Due to the rare occurrence of Grisel’s syndrome, our review is limited to case reports only, and this may limit the generalizability of our findings due to the smaller sample size; moreover, the diagnostic and management strategies employed are to the best of the knowledge and experience of authors in their practice and single-center setting only, hence this further limit the generalizability, which, however, additionally highlights the dearth of literature in this aspect; therefore, further research can be beneficial in the development of standardized management guidelines for practice to ensure optimal outcomes and prevent complications.

## Conclusions

In children who present with acquired torticollis, Grisel's syndrome should be taken into consideration, especially if there has been a previous otolaryngological procedure or infection. Treatment options include muscle relaxants, soft collars, cervical traction, or even C1 and C2 arthrodesis, depending on the clinical findings and Fielding classification of the degree of the subluxation. Early diagnosis and prompt management are essential in Grisel's syndrome since delay can lead to neurological and fatal complications. Additionally, the prognosis is excellent if the condition is identified early and treated appropriately. Clinical research in the future involving a significant study/target population is critical for the development of generalized evidence-based management strategies and can additionally aid in defining preventive guidelines for patients undergoing otolaryngology procedures.
